# MicroRNA levels quantified in whole blood varies from PBMCs

**DOI:** 10.12688/f1000research.4884.4

**Published:** 2015-10-06

**Authors:** Sadaf Atarod, Hannah Smith, Anne Dickinson, Xiao-Nong Wang

**Affiliations:** 1 Haematological Sciences, Institute of Cellular Medicine, Newcastle University, Newcastle upon Tyne, NE2 4HH, UK

**Keywords:** MicroRNAs, PBMC

## Abstract

MicroRNAs are non-coding RNAs that negatively regulate mRNA expression and play significant roles in both health and disease. Differential microRNA expression has been used to aid diagnosis and discriminate disease stages. The accuracy and reliability of microRNA expression measurement is of utmost importance. Quantification of microRNA expression in human peripheral blood is commonly detected using total RNA extracted via different methods. To date, no convincing data are available showing whether microRNA quantification results can be influenced by the use of total RNA extracted from whole blood or peripheral blood mononuclear cells (PBMCs). This study examined miR-146a-5p and miR-155-5p expression using total RNA extracted in parallel from whole blood and PBMCs of 14 healthy volunteers. The data showed that the quantification of miRNA using total RNA extracted from whole blood varied from that of PBMCs, indicating that the miRNA expression was a result of all the different cell-types present in whole blood. Our results suggested that the source of total RNA and the statistical analyses performed are crucial considerations when designing miRNA research.

## Introduction

MicroRNAs (miRNAs) are cell- and therefore tissue-specific, and their expression levels impact protein translation ( Sood *et al.*, 2006). Nearly, 2000 microRNA (miRNA) sequences have been identified in humans ( Kozomara & Griffiths-Jones, 2014). Numerous studies have reported specific miRNA expression levels in peripheral blood (PB) as markers of disease ( Mookherjee & El-Gabalawy, 2013; Patnaik *et al.*, 2012). Although miRNA expression levels could be inevitably influenced by the way that total RNA is extracted, often studies reporting differential miRNA expression levels fail to emphasise the impact of RNA extraction methods. This could at least partially lead to significant controversies and inconsistencies in the literature related to miRNA research.

PAXgene Blood RNA System (PAXgene Blood RNA Tube and PAXgene Blood miRNA Kit [PAXM]) has been the gold standard for PB collection as the stabilising reagent present in the tube prevents RNA degradation and inhibits changes in gene expression due to the collection procedure ( Viprey *et al.*, 2012). However, it should be emphasised that the miRNA expression detected in whole blood is the overall outcome from total hematocytes rather than the lymphocyte fraction only. Recent studies have shown that erythrocytes also contain a high proportion of miRNAs ( Hamilton, 2010). Bayatti *et al.* have shown that the presence of globin (globular proteins such as haemoglobin) in PB can impact RNA expression and that globin depletion can decrease total RNA quality and yield in particular when extraction is performed using the PAXgene Blood RNA kit ( Bayatti *et al.*, 2014). However, it is necessary to evaluate the miRNA expression levels in an independent cohort, as miRNAs are in general highly stable, and therefore their expression might not be affected by globin treatment. A recent research has reported that miRNA expression levels detected using whole blood correlated to that of PBMCs when using PAXgene Blood RNA System and *mir*Vana miRNA kit (MM), respectively ( Mookherjee & El-Gabalawy, 2013). The study did not show that the expression levels are comparable or that they agree with each other using the recommended Bland-Altman method comparison statistical test ( Bland & Altman, 1986). This has raised some confusion as the PAXgene Blood RNA system extracts from whole blood while MM from PBMCs. Performing a comprehensive statistical analysis is required when two methods are compared ( Burd, 2010).

**Figure 1.**
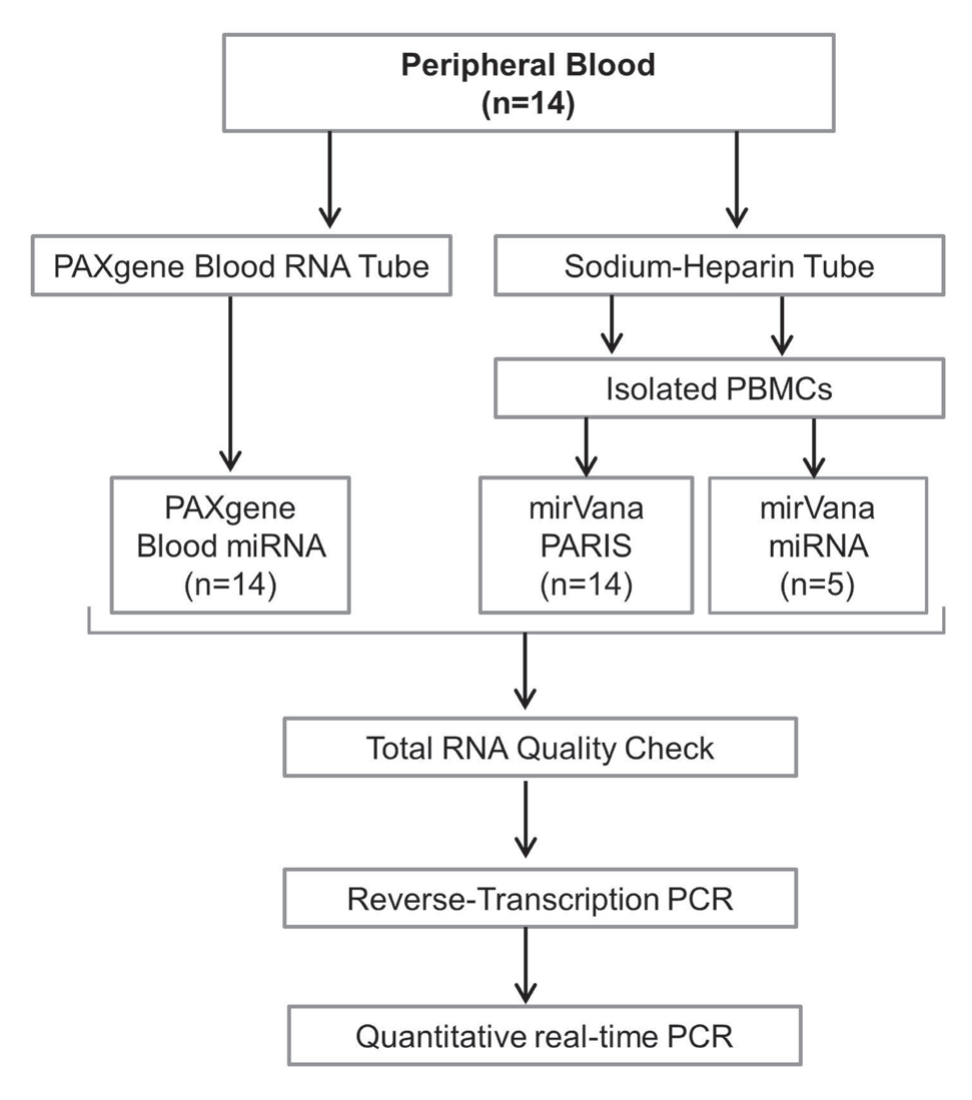
Experimental design. Peripheral blood was collected into various tubes as illustrated. Total RNA was extracted from whole blood using the PAXgene blood miRNA kit and from PBMCs using the *mir*Vana Paris and *mir*Vana isolation kits, respectively. The total RNA contained the miRNA population which was measured by firstly performing reverse transcription and then quantitative real-time PCR.

In this investigation we have measured the expression of miR-146a-5p and miR-155-5p which have extensively been investigated in whole blood and PBMCs due to their critical functions in the innate and adaptive immune system ( Curtale *et al.*, 2010; Schulte *et al.*, 2013). We sought to compare miRNA expression levels in peripheral blood collected from 14 healthy volunteers using both extraction methods (PAXM and *mir*Vana PARIS [MP]). We sought to address (i) whether miRNA expression detected in whole blood is comparable to that of isolated PBMCs and (ii) to detect the presence of haemolysis in whole blood and PBMCs. The experimental design for this investigation is depicted in Figure 1.

## Materials and methods

### Volunteer cohort

Whole blood was collected from 14 healthy volunteers following approval from the Newcastle and North Tyneside 2 Research Ethics Committee (STEMDIAGNOSTICS: REC-07/H0906/131) and informed consent was obtained from every volunteer for both blood collection and miRNA testing. Samples from 14 healthy volunteers (5 males and 9 females) were used to quantify miRNA expression in whole blood and PBMC.

### Sample collection and storage

PB (2.5 ml) was collected in PAXgene Blood RNA Tubes (PreAnalytiX GmbH, Switzerland [Catalog No: 762165]) containing an RNA stabilising agent that lysed the blood cells and stabilised the intracellular RNA. The tubes were stored at -20°C until processed. PB was also collected in sodium (Na)-heparin (Sigma, UK) containing tubes for peripheral blood mononuclear cell isolation using graduated centrifugation over Lymphoprep™ (STEMCELL Technologies, Manchester, UK). The cells were then stored on ice before extraction. Isolated PBMCs were cryopreserved by re-suspension in freezing solution containing 70% RPMI 1640 (Sigma-Aldrich, UK), 20% fetal calf serum and 10% dimethyl sulfoxide (NBS Biologicals, UK) and stored in Cryovials at -80°C.

### Total RNA extraction from peripheral blood

Total RNA was extracted from isolated PBMCs using the (i) *mir*Vana™ PARIS™ kit (MP) and (ii) *mir*Vana™ miRNA Isolation kit (MM) (Ambion, USA [Catalog Nos: AM1556 and AM1560, respectively]) according to the manufacturer’s protocol. PAXgene Blood RNA Tubes were incubated overnight at room temperature to increase the RNA yield. Total RNA extraction from whole blood was performed using the PAXgene Blood miRNA kit (PAXM) (PreAnalytiX GmBh, Switzerland [Catalog No: 763134]), according to the manufacturer’s protocol. Aseptic techniques were followed at all stages of extraction. Total RNA quality and concentration were assessed using NanoDrop ND-1000 spectrophotometer (Thermo Fischer Scientific, MA). The absorbance ratios 260 nm/280 nm and 260 nm/230 nm were analysed to determine the purity of total RNA.

### Reverse transcription and quantitative real-time PCR

MicroRNA specific cDNA was synthesised from 10 ng total RNA extracted from isolated PBMCs and whole blood using TaqMan MicroRNA reverse transcription (RT) kit (Applied Biosystems, Life Technologies, USA [Catalog No: 4366596]) as per the manufacturer’s protocol. The same RNA concentration was used for all the reactions. Hydrolysis probes were used for cDNA synthesis (Assay IDs: miR-155-5p-5p: 000479, miR-146a-5p: 000468, miR-451-5p: 001141, miR-23a-3p: 000399, SNORD49A [RNU49]: 001005, SNRNP27 [U6]: 001973, SNORA74A [RNU19]: 001003 and SNORD48 [RNU48]: 001006). No-enzyme control (NEC) and a negative template control (NTC) were run for every extraction and RT reaction set. The samples were then run on a thermal cycler at four different holding temperatures: 16°C for 30 minutes, 42°C for 30 minutes, 85°C for 5 minutes and finally at 4°C until storage at -4°C.

Quantitative real-time PCR (qPCR) was performed using the TaqMan method and hydrolysis probes mentioned above (Applied Biosystems by Life Technologies, CA, USA) according to the manufacturer’s protocol. Each sample was run in triplicate and every plate contained the NTC from the RT step and the qPCR step as well as NEC on a 7900HT Fast Real-Time PCR System (Life Technologues, CA, USA).

### Data analysis

The qPCR results were analysed using SDS v2.4 software and normalised using SNORD48 as the reference control which was selected by testing a panel of four controls for stable expression within the whole blood and PBMCs. The comparative ∆∆C _q_ method was used to calculate fold-changes (ΔC _q_ = C _q microRNA of interest_ - C _q reference control_, Relative Quantification [RQ]=2 ^-ΔΔCq^ and LOG transformed = LOG _2_RQ). Fold-change was logarithm-transformed as qPCR data are non-linear (exponential), and is transformed to decrease the heterogeneity of variance ( McDonald, 2009) and also to identify the outliers present in the data ( Rieu & Powers, 2009). Standard curves for three samples from both PAXM and MP were generated and a 95% confidence interval slope of the line was used to calculate the PCR efficiency (E) using the standard formula; E=10 ^-1/Slope^ and % efficiency = (E-1) × 100. Mean efficiency was then calculated. Results were analysed and plotted using GraphPad PRISM v5.0 software (GraphPad Software, Inc, USA). Mann-Whitney U t-test was used to assess difference between two groups and Kruskal-Wallis one-way analysis of variance (ANOVA) for multiple groups. Spearman’s test was used to determine correlation. Bland-Altman was performed to test whether two methods agreed and if one could be interchanged with another. Significance was set at p<0.05.

## Results

### Quality of RNA extracts

**Figure 2.**
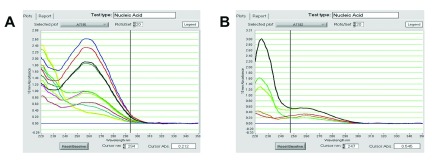
Representative graphical plots of total RNA extracted. ( **A**) PAXM: analysis of whole blood showed peaks positioned at 230 nm (n=10) and ( **B**) MP: analysis of PBMCs showed peaks positioned at 260 nm (n=5).

Total RNA was extracted using three different extraction methods; PAXM (PAXgene Blood miRNA), MP (mirVana PARIS) and MM (miRVana miRNA) from whole blood and PBMCs. RNA purity was assessed by detecting the absorbance ratios at 260 nm/280 nm and at 260 nm/230 nm. The ratio (260 nm/280 nm) for whole blood was 1.95 – 2.35 and for isolated PBMCs was 2.00 – 2.27 (both MP and MM respectively). The ratios were ≥ 1.8 – 2.0; thus extraction was free from protein contamination which is usually absorbed at 280 nm. Absorbance ratios detected at 260 nm/230 nm showed ratios below the accepted contamination-free range of 1.5 – 2.0 (PBMCs: 0.18 – 1.83 and whole blood: 0.15 – 1.49). Therefore, the samples may have been affected by contaminants absorbed at 230 nm such as guanidine isothiocyanate present in all the three extraction kits. For RNA purity, the peak of each total RNA plot was also analysed as it could indicate contamination by phenol and/guanidine isothiocyanate. PBMCs had plot peaks at 260 nm thus confirming contaminant-free samples. However, peaks at 260 nm were absent for total RNA extracted using the PAXM ( Figure 2). This further suggests that guanidine salts may have been the cause of contamination in whole blood samples extracted using PAXM.

### Quantitative PCR quality controls

**Supplementary Figure 1.**
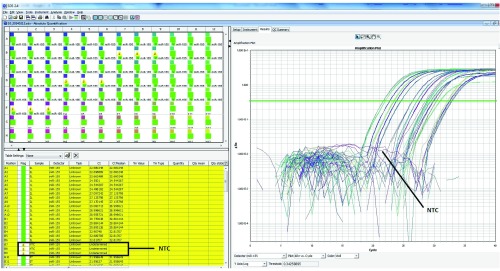
RT-qPCR amplification curves. There was no amplification observed in the no-template control (NTC) sample.

NEC and NTC controls were used to ensure that the RT and qPCR reactions were contaminant-free. Each control displayed no amplification (Cq > 36). This was particularly important for total RNA extracted from PBMCs, as they were non-DNase treated. Thus, any amplification in the controls may have suggested either non-specific binding of primers or presence of contamination such as genomic DNA. In addition we calculated the average efficiency of our real-time qPCR reactions (E=97.8%) which confirmed absence of reaction inhibitors such as heparin that was specifically used for the collection of peripheral blood from patients for the downstream PBMC isolation. (See Supplementary Figure 1, that shows there was no contamination in the samples with a Cq value equal to 40 i.e. no amplification). Determining qPCR efficiency is important as inhibitory compounds can affect miRNA expression and result in false positives.

### Reference control for microRNA expression in peripheral blood

**Figure 3.**
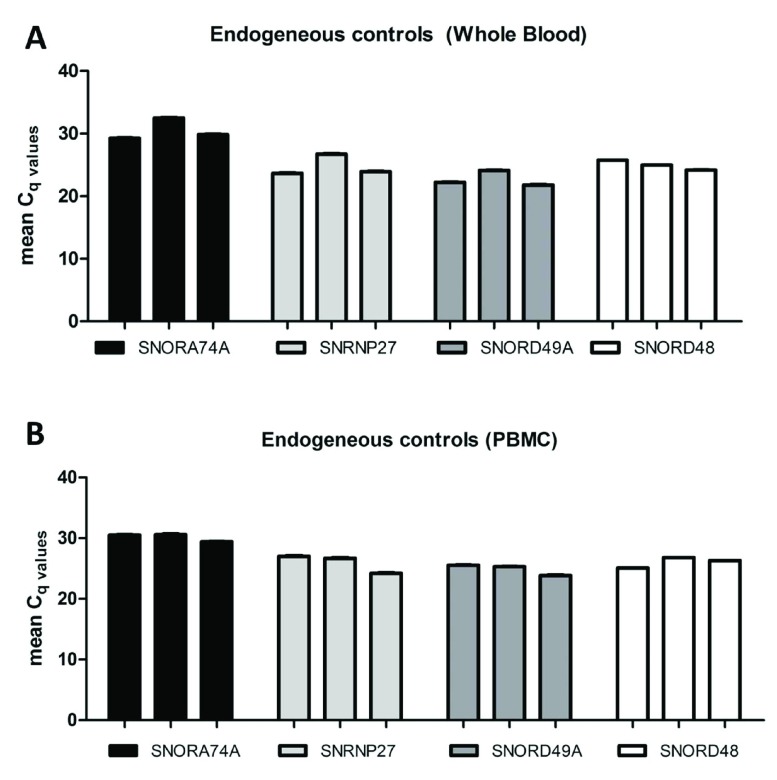
Expression of endogenous controls in total RNA extracted. A panel of four stably expressed miRNAs were selected and quantified to identify the most stable control for normalisation of miR-146a-5p and miR-155-5p in paired samples (n=3) ( **A**) whole blood and ( **B**) PBMCs. The standard error of the mean is shown by the error bars for demonstration of technical variability.

We determined the most appropriate reference gene for this investigation by testing a panel of four controls that have been known for stable expression (SNORD49A [RNU49], SNRNP27 [U6], SNORA74A [RNU19] and SNORD48 [RNU48]) ( Dataset a). Our results showed that SNORD48 expression was the most stable in total RNA extracted via both the PAXM ( Figure 3A) and MP ( Figure 3B) method. Therefore, SNORD48 was used as the reference control to normalise miR-146a-5p and miR-155-5p expression in each sample.

### Whole blood extracted using PAXM exhibits higher degree of haemolysis

**Figure 4.**
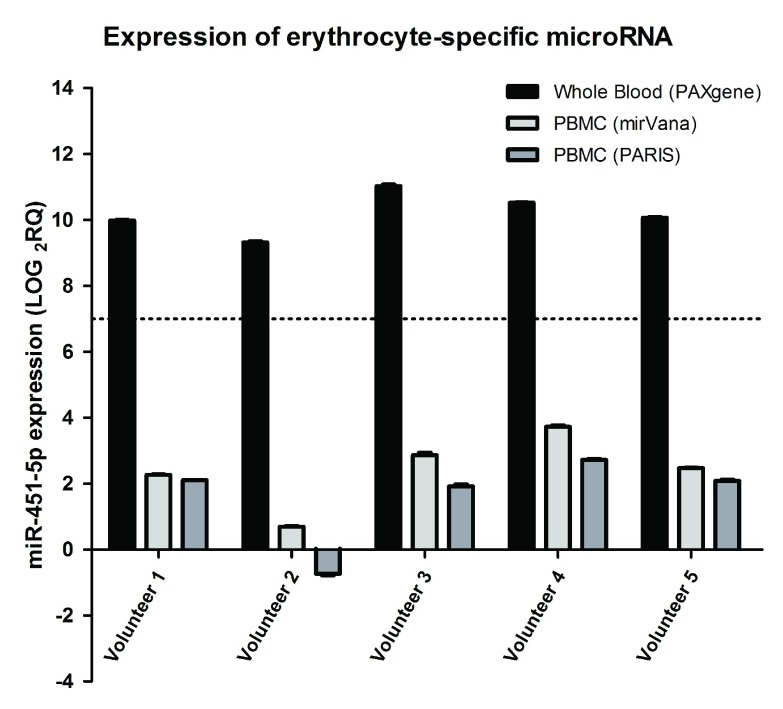
Degree of haemoloysis in total RNA extracted via the three extraction methods. Total RNA extracted using the PAXgene miRNA isolation kit. A threshold of LOG _2_RQ greater than 7 was indicative of haemolysis (dashed line). The degree of haemolysis was calculated by measuring the difference between miR-451-5p and miR-23a-3p expression. All data (n=5) have been log-transformed. The standard error of the mean is shown by the error bars for demonstration of technical variability.

Erythrocyte haemolysis has been reported to alter miRNA measurements in whole blood, plasma, serum and tissues ( McDonald *et al.*, 2011; Pritchard *et al.*, 2012). The total RNA extracted using the three different methods was examined for degree of haemolysis by quantifying miR-451-5p and miR-23a-3p ( Dataset b). Normalised ∆Cq values (miR-23a-3p – miR-451-5p) greater than seven were considered as an indicator of haemolysis. Our results showed that there was a significantly high degree of haemolysis in the total RNA extracted using the PAXM method with the ∆Cq values in the range of 9 – 11. Haemolysis was low in total RNA extracted using either of MP or MM, ∆Cq>3 ( Figure 4).

### MicroRNA expression in whole blood and PBMCs

**Figure 5.**
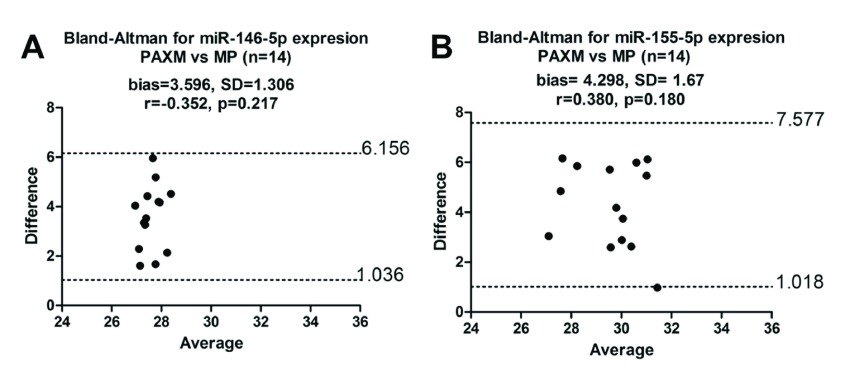
Bland-Altman plots for miRNA expression from whole blood and PBMCs. Total RNA was extracted (n=14) using PAXM and MP for ( **A**) miR-146a-5p and ( **B**) miR-155-5p expression. MicroRNA expression is within the limits of agreement but the bias is greater than one showing high disagreement between PAXM and MP. r indicates Spearman correlation. SD: Standard Deviation and bias is the mean difference. C _q_ values were used for this analysis. Dashed lines show the 95% lower and upper limits of agreement.

**Figure 6.**
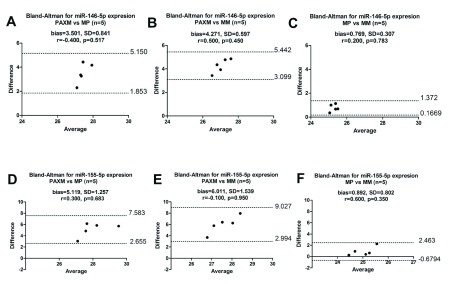
Bland-Altman plots for miR-146a-5p and miR-155-5p expression in whole blood and PBMCs. The three methods were all compared for miR-146a-5p as ( **A**) PAXM vs MP ( **B**) PAXM vs MM and ( **C**) MP vs MM as well as miR-155-5p ( **D**) PAXM vs MP ( **E**) PAXM vs MM ( **F**) MP vs MM. MicroRNA expression is within the limits of agreement but the bias is greater than one showing high disagreement between PAXM and MP. Bias is lower than one for MP and MM, thus the two methods agree with one another. r indicates Spearman correlation. SD: Standard Deviation and bias is the mean difference. C _q_ values were used for this analysis. Dashed lines show the 95% lower and upper limits of agreement.

**Figure 7.**
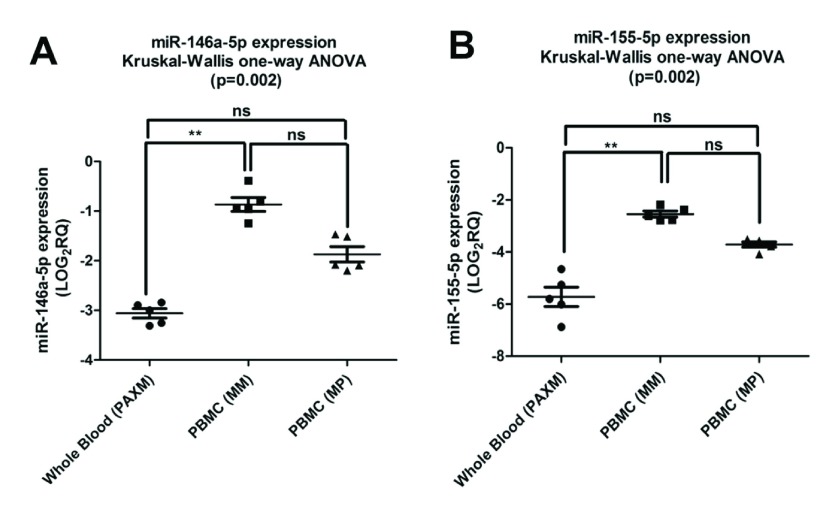
Normalised miRNA expression comparison from whole blood and PBMCs. ( **A**) miR-146a-5p and ( **B**) miR-155-5p expression. MicroRNA expression is significantly varied across all the three different groups (p=0.002). MicroRNA expression is higher in PBMCs extracted via either MP or MM method in comparison to whole blood. **p<0.01 and ns: not significant.

With inclusion of stringent quality controls, we assessed miR-146a-5p and miR-155-5p expression in total RNA extracted in parallel from whole blood using PAXM (n=14) and PBMCs using MP (n=14) ( Dataset c). Our results showed that there was no correlation ( Figure 5, miR-146a-5p: r=-0.352, p=0.217 and miR-155-5p: r=0.380, p=0.180) between PAXM and MP in the expression of both miR-146a-5p and miR-155-5p. In a PCR reaction, it is assumed that the target expression doubles at every reaction cycle. Bland-Altman analysis also showed that the two methods did not agree as the bias was greater than 1 which equated to more than one qPCR cycle difference between the two methods ( Figure 5A and 5B). Mookherjee *et al.* had used MM to extract total RNA from PBMCs, then correlated miRNA expression between the PAXM and MM method ( Mookherjee & El-Gabalawy, 2013). To eliminate the possibility that using MP was the reason for the non-correlation and disagreement, we tested the three different extraction methods (PAXM, MP and MM) for both miRNAs in a randomly selected cohort of five healthy volunteers ( Figure 6) ( Dataset d). The results demonstrated that PAXM and MP as well as PAXM and MM did not correlate nor agreed with one another. However, MP and MM methods agreed with each other and could therefore be interchanged as the bias between the two methods for both miR-146a-5p and miR-155-5p was only 0.769 (SD=0.307) and 0.892 (SD=0.802), respectively. Interestingly, normalised miRNA expression was significantly different only between PAXM and MM methods (miR-146a-5p and miR-155-5p: p<0.01). There was higher miRNA expression in PBMCs than in whole blood for both miRNAs ( Figure 7).

Data of miRNA extraction methods from whole blood and PBMCs Dataset (a) shows the expression of all endogenous controls used in the study. Dataset (b) shows the degree of cell hemolysis using different extraction kits. Datasets (c)-(d)-(e) contain data of miR-146a-5p and miR-155-5p expression in whole blood and PBMCs in different samples. Complete dataset legends can be found in the text file. Click here for additional data file. Copyright: © 2015 Atarod S et al. 2015 Data associated with the article are available under the terms of the Creative Commons Zero "No rights reserved" data waiver (CC0 1.0 Public domain dedication).

## Discussion

 MicroRNA expression levels are used to classify diseases and also to distinguish the diseased from the healthy population. However, lack of uniform detection protocols has led to controversies and inconsistencies in miRNA research. There is also lack of recognition for the presence of miRNAs from erythrocytes and other cell-types when using whole blood for total RNA extraction processes and downstream miRNA studies. In most investigations, PBMCs are considered as the major cellular sources for miRNAs. This work was conducted to elucidate the difference between total RNA extracted from whole blood and PBMCs for miRNA expression level studies and also to highlight the importance of protocol standardization.

RT-qPCR was performed to examine whether the expression of miR-146a-5p and miR-155-5p in whole blood and PBMC agreed with one another. Our results showed that there was no agreement between PAXM and both MP and MM for miR-146a-5p and miR-155-5p expression. PBMCs constitute only a fraction of the cells present in PB and therefore lack granulocytes, platelets and erythrocytes ( Min *et al.*, 2010). Due to the unique miRNA expression pattern in each cell-type the relative proportions of cells in blood may have an effect on the overall miRNA expression profile and the expression of their protein targets ( Min *et al.*, 2010). Some studies have shown that mature miRNA expression signature in erythrocytes is similar to that in whole blood while different when compared to PBMCs ( Chen *et al.*, 2008). MiR-451-5p is a marker of erythrocytes ( Rasmussen *et al.*, 2010) and miR-23a-3p is unaffected by haemolysis ( Blondal *et al.*, 2013). We have shown that there is lower miR-146a-5p and miR-155-5p expression in whole blood compared to PBMCs demonstrating that the total RNA extracted from PBMCs does not reflect that detected in whole blood. Several studies have shown that total RNA yield from whole blood decreases after the use of DNase step in the PAXM protocol ( Asare *et al.*, 2008; Bayatti *et al.*, 2014; Debey *et al.*, 2004). However, if PAXM total RNA is not DNase-treated, there may be a possibility of DNA contamination. Contrary to our results, Mookherjee *et al.* found a linear correlation between miR146a-5p and miR-155-5p expression in whole blood and isolated PBMCs collected from a healthy population ( Mookherjee & El-Gabalawy, 2013). In their work, they did not measure the degree of haemolysis in the samples, which may partly explain the discrepancy between the two studies. Furthermore, our study compared both the strength (correlation) and level of agreement between the two methods whilst Mookherjee *et al.* examined only the correlation ( Bland & Altman, 1986). This highlights the importance of performing the correct statistics when two methods are compared with regards to their equivalence and interchangeability ( Burd, 2010).

 In clinical practice, it is easier to collect PB in PAXgene Blood RNA tubes as they have a shelf-life of two to five years without any RNA degradation. Immediate stabilisation is vital as storage of blood cells induce changes in the miRNA composition ( Gaarz *et al.*, 2010). Thus, extraction methods from whole blood must be optimised to either eliminate erythrocyte contamination or consider the expression as cumulative and design downstream experiments for miRNA protein target studies accordingly.

In conclusion, our study showed differences in miR-146a-5p and miR-155-5p expression in isolated PBMCs and whole blood. We suggest that PBMCs are not the ideal source to study and correlate miRNA protein targets where the miRNA expression had been measured in whole blood as the miRNA expression pattern in whole blood is not comparable to that in PBMCs. We also highlight the importance of having a stringent set of technical controls and performing the correct statistics to increase the reliability and reproducibility of miRNA expression studies.

## Consent

All participants to the study provided informed written consent for molecular testing and publication of the data.

## Data availability

The data referenced by this article are under copyright with the following copyright statement: Copyright: © 2015 Atarod S et al.

Data associated with the article are available under the terms of the Creative Commons Zero "No rights reserved" data waiver (CC0 1.0 Public domain dedication). 

*F1000Research*: Dataset 1. Data of miRNA extraction methods from whole blood and PBMCs, 10.5256/f1000research.4884.d33496 ( Atarod *et al.*, 2014).
