# Supplementary material for: MicroRNA levels quantified in whole blood varies from PBMCs
Source: F1000Res. 2015 Oct 6;3:183. Originally published 2014 Aug 5. [Version 4] doi: 10.12688/f1000research.4884.4 (PMC4582824; doi:10.12688/f1000research.4884.4)
Supplement: Peer review history

# Peer review: Referee response for version 3

**DOI:** 10.5256/f1000research.7159.r10479

**Published:** 2015-09-24

**Authors:** Stefano Casola

**Affiliations:** 1 Genetics and Epigenetics of Lymphocyte Development and Transformation Research Program, Institute of Molecular Oncology Foundation (IFOM Institute), Milan, Italy

In this manuscript, Atarod and colleagues quantify the expression levels of two candidate miRNAs, mir-146-5p and mir-155-5p, starting from either whole blood (WB) or PBMC.  Authors employ quantitative real-time PCR to measure candidate miRNA transcripts from the two biological sources. Different procedures were used to extract total RNA from the two sources, which could, at least in part, affect downstream expression analyses.  The study is sound and the data are convincing and relevant for appropriate selection of the blood source from which to measure levels of miRNAs of interest. The conclusions reached by the authors indicate poor correspondence in the expression of mir-146-5p and mir-155-5p when comparing WB to PBMC as biological source. The results by Atarod and colleagues are in apparent contrast with those described in a previous study by Mookherjee & El-Gabalawy. The reasoning to explain the discrepancy is convincing, and raises important issues related to technical (grade of haemolysis) and methodological aspects (statistical analysis) of the expression analysis that could undermine a correct interpretation of the data.

The title is appropriate and data are presented and discussed in a concise and clear fashion.

The manuscript has benefited from previous rounds of revision, addressing the main concerns raised by the reviewers.

Minor points:

Materials and methods section:

1. Sample collection and storage: Authors state that, after purification, PBMCs were stored at -4 **°**C. Is that correct? Or, instead it should read +4°C?
2. Authors state “ *PBMC were cryopreserved by re-suspension in freezing solution containing 350 **ml **of RPMI 1640..” *Authors should replace “350 ml” with (I suspect) 350µl.

I have read this submission. I believe that I have an appropriate level of expertise to confirm that it is of an acceptable scientific standard.

# Response

**Published:** 2015-09-25

**Authors:** Sadaf Atarod

**Affiliations:** Newcastle University, UK, UK

Thank you for reviewing our manuscript and your valuable comments. Please, find below our replies.

1. We stored the cells on ice, as the extraction was performed soon after the collection. This information has been updated in the revised manuscript.

2. We have updated the revised manuscript with the percentage of each ingredient. We used 70% RPMI to make up the freezing solution.

# Peer review: Referee response for version 3

**DOI:** 10.5256/f1000research.7159.r9135

**Published:** 2015-06-23

**Authors:** Steven O'Reilly

**Affiliations:** 1 School of Biological and Biomedical Sciences, Durham University, Durham, UK

In light of the new version and the author’s response to my previous review, I am happy to approve this article.

I have read this submission. I believe that I have an appropriate level of expertise to confirm that it is of an acceptable scientific standard.

# Peer review: Referee response for version 2

**DOI:** 10.5256/f1000research.6331.r8664

**Published:** 2015-05-14

**Authors:** Steven O'Reilly

**Affiliations:** 1 School of Biological and Biomedical Sciences, Durham University, Durham, UK

This is a very interesting an important study that highlights differences in microRNA expression between whole blood and isolated Peripheral Blood Mononuclear Cells (PBMCs). This is of clear importance where as many authors would be using whole blood microRNA expression and then using isolated PBMCs to draw conclusions on their putative targets. There is clear differences between the two due to the fact of other sources of microRNAs in the whole blood including erythrocytes and platelets and the fact that there are also ‘free floating’ microRNAs in blood also, likely encased in exosomes. This study is technically sound and I particularly like the fact that the authors calculated the qPCR efficiency. The design is also technically sound.

I would suggest that the authors include a ‘screen shot’ of the actual amplification curve of the No Template Control (NTC).  This will confirm visually that there is no contamination in the samples with a likely CT value 37>.

Did the authors after lymphoprep isolation notice any difference in expression of the microRNAs before and after cryopreservation?  The cryopreservation medium contains DMSO (although low) and we have found this affects certain microRNA expression patterns.

Was there any apparent difference in the levels of the microRNAs correlation with age? I.e. old donors had lower or higher levels of miR-146a because they are immunosenescent (but not diseased)?

I have read this submission. I believe that I have an appropriate level of expertise to confirm that it is of an acceptable scientific standard, however I have significant reservations, as outlined above.

# Response

**Published:** 2015-06-12

**Authors:** Sadaf Atarod

**Affiliations:** Newcastle University, UK, UK

Thank you for reviewing our manuscript and your valuable comments. Please, find below our replies.

1. I would suggest that the authors include a ‘screen shot’ of the actual amplification curve of the No Template Control (NTC). This will confirm visually that there is no contamination in the samples with a likely CT value 37>.

Thanks for the suggestion. This figure has been added as a supplementary figure to the manuscript.

2. Did the authors after lymphoprep isolation notice any difference in expression of the microRNAs before and after cryopreservation? The cryopreservation medium contains DMSO (although low) and we have found this affects certain microRNA expression patterns.

We have evaluated the impact of cryopreservation on miRNA expression by extracting total RNA from paired fresh and cryopreserved PBMCs (n=5) and performed RT-qPCR respectively. There was no significant difference between fresh and cryopreserved PBMCs for the expression of miR-146a-5p and miR-155-5p (both p=0.125). For the figure, please refer to the first version of this paper. In addition, there was no correlation in miRNA expression between fresh and cryopreserved PBMCs with the bias of <1 for both miR-146a-5p and miR-155-5p.Thus, in this study, expressions of miR-146a-5p and miR-155-5p in fresh and cryopreserved PBMCs agreed with one another although further studies including more samples and longer period of cryopreservation may provide more informative evidence. Moreover, this finding may not be generalised to other miRNAs as individual miRNAs may have varied susceptibility to cryopreservation.

3. Was there any apparent difference in the levels of the microRNAs correlation with age? I.e. old donors had lower or higher levels of miR-146a because they are immunosenescent (but not diseased)?

In this investigation, there was no correlation between age and miRNA expression levels. However, this investigation cohort included only three older volunteers (age> 55 years). Thus, this finding requires further confirmation in a larger cohort of samples and with older age.

# Peer review: Referee response for version 1

**DOI:** 10.5256/f1000research.5214.r6354

**Published:** 2014-10-17

**Authors:** Kenneth Witwer

**Affiliations:** 1 Molecular and Comparative Pathobiology, Johns Hopkins University, Baltimore, MD, USA

The design of this study is flawed, and several conclusions are not justified by the results. Since the PCR technique seems sound (and the authors are commended for including information such as PCR efficiency), there may be several subcomponents of the study that could be separated out and published as a smaller paper (e.g., the results of the gender and freezing experiments).

1. I would encourage the authors **to remove immediately the baseless claims about putative problems with the PAXgene reagents or protocol. A major (and wrong) conclusion of the paper is that the PAXgene method results in putatively excessive hemolysis, or, as the Figure 4 title incorrectly states, "Erythrocyte contamination". The putative difference from the other methods is described as "significantly" high, and although no statistical analysis is reported, one could hardly quibble with a ten-order-of-magnitude difference. However, this difference has nothing to do with hemolysis as a processing variable and in fact reflects well, not poorly, on the PAXgene method. The PAXgene tube is designed to lyse blood cells and stabilize RNA. This study is thus not an examination of methods, hemolysis or RBC contamination! It is simply a confirmation that lysed RBCs contain RBC RNA, whereas PBMCs do not contain RBC RNA.
2. There appears to be a fundamental confusion about what constitutes hemolysis vs. red blood cell contamination. Where is the evidence that "haemolysis has been reported to alter miRNA measurements in whole blood, plasma, serum, and tissues"? The two cited references examine the effects of hemolysis in plasma and serum, where lysis of cells can indeed have a great effect. It is clear that the very low abundance extracellular RNA fraction of blood is influenced by an influx of cellular RNA. It is unclear how degree of hemolysis could affect detection of very high abundance cellular miRNAs in whole blood (where erythrocytes dominate anyway) or in cells like PBMC that are purified away from other components.
3. The title does not describe the study. The study is presented incorrectly as a comparison of RNA extraction methods and of RNA expression in whole blood and PBMCs. Unfortunately, too many variables are changed at once in the study to allow any conclusions to be drawn. For example (see Figure 1): substrate type (whole blood vs PBMC); initial treatment (PAXgene tube for whole blood, heparin for PBMC); cell isolation (none for whole blood, centrifugation over a gradient for PBMC); and RNA isolation method (PAXgene for whole blood, two nearly identical Ambion kits for PBMC). The only variable that can be isolated is the use of the mirVana PARIS vs miRNA kit.
4. Heparin, as a PCR inhibitor, may not be the best anticoagulant for use in qPCR-based studies. No special steps are described for removal of heparin.

Minor points

1. There are no error bars to indicate biological or technical variability in Figure 3 or 4.
2. The workflow of Figure 1 does not indicate that the right-hand-side pathway involved only PBMC, not whole blood.
3. The PAXgene Blood RNA Tube and the PAXgene Blood miRNA Kit are presented as the "gold standard" in the field, yet the citation is a publication by the company, and it does not mention the miRNA kit. This should be replaced by a more objective assessment.
4. More clarity is needed in the use of terms. For example, there may or may not be RNA "expression" differences between genders or cell types. In contrast, differences in RNA quantitation observed upon freezing, or addition of PCR inhibitors, or after purification of the same sample with different kits, are not due to "expression" differences. The underlying expression in the cell remains the same; the ability to characterize it changes.
5. Citations of the literature are somewhat thin.

I have read this submission. I believe that I have an appropriate level of expertise to state that I do not consider it to be of an acceptable scientific standard, for reasons outlined above.

# Response

**Published:** 2014-12-01

**Authors:** Sadaf Atarod

**Affiliations:** Newcastle University, UK, UK

Thanks for the valuable comments. We admit full heartedly that some descriptions and statements in the text were not as clear as they should be. This has caused misunderstanding and confusion.  Good efforts have been given to improve the clarity of the text and to address each comment by the reviewer. We hope the revised manuscript could convince the reviewer (and the readers) that the design of the study is sound and rational and the conclusions can be well justified by the results.

As suggested, the results of gender and cryopreservation experiments have been excluded to deliver a more focused message.

The title of the manuscript has also been changed to clarify the message of the

 

1. *"I would encourage the authors to remove immediately the baseless claims about putative problems with the PAXgene reagents or protocol. A major (and wrong) conclusion of the paper is that the PAXgene method results in putatively excessive hemolysis, or, as the Figure 4 title incorrectly states, "Erythrocyte contamination". The putative difference from the other methods is described as "significantly" high, and although no statistical analysis is reported, one could hardly quibble with a ten-order-of-magnitude difference. However, this difference has nothing to do with hemolysis as a processing variable and in fact reflects well, not poorly, on the PAXgene method. The PAXgene tube is designed to lyse blood cells and stabilize RNA. This study is thus not an examination of methods, hemolysis or RBC contamination! It is simply a confirmation that lysed RBCs contain RBC RNA, whereas PBMCs do not contain RBC RNA*." 
  This article has no intention at all to claim any problems with the PAXgene protocol or to examine haemolysis or RBC contamination. The key points we intended to raise in this manuscript are 1) the use of total RNA extracted from whole blood or isolated PBMC could lead to differential results of miRNA quantification as miRNAs expressed in erythrocytes are present in total RNAs extracted from whole blood but not isolated PBMCs; 2) This inevitable fact is partly accountable for many conflicting reports in the existing literatures; 3) This is an important factor that researchers should consider when conducting miRNA research so as to extract total RNAs from most appropriate starting material (whole blood or isolated PBMC). Particularly, total RNAs extracted from the same starting material should be used for analysis of specific miRNAs and their downstream protein targets. These points have been clarified in the revised manuscript.
  Furthermore, Figure 4 was intended to demonstrate the fact that erythrocyte related miRNA is present and detectable when using total RNAs extracted from whole blood (not to show red blood cell contamination). The title of Figure 4 and the related contents in the text have been revised to clarify this point that it is not a contamination *per se *but a factor that needs to be considered when analysing, reporting results and investigating miRNA protein targets.
2. *"There appears to be a fundamental confusion about what constitutes hemolysis vs. red blood cell contamination. Where is the evidence that "haemolysis has been reported to alter miRNA measurements in whole blood, plasma, serum, and tissues"? The two cited references examine the effects of hemolysis in plasma and serum, where lysis of cells can indeed have a great effect. It is clear that the very low abundance extracellular RNA fraction of blood is influenced by an influx of cellular RNA. It is unclear how degree of hemolysis could affect detection of very high abundance cellular miRNAs in whole blood (where erythrocytes dominate anyway) or in cells like PBMC that are purified away from other components."*
  We believe this is a confusion caused by the inappropriate use of terminology rather than a flaw in study design. The revised manuscript has clarified these points. The discussion point of the manuscript is neither about haemolysis nor red blood cell contamination but simply about the fact that the presence of red blood cells in whole blood method may give rise to different miRNA quantification results compared to those where isolated PBMCs were used to extract total RNAs.
3. *"The title does not describe the study. The study is presented incorrectly as a comparison of RNA extraction methods and of RNA expression in whole blood and PBMCs. Unfortunately, too many variables are changed at once in the study to allow any conclusions to be drawn. For example (see Figure 1): substrate type (whole blood vs PBMC); initial treatment (PAXgene tube for whole blood, heparin for PBMC); cell isolation (none for whole blood, centrifugation over a gradient for PBMC); and RNA isolation method (PAXgene for whole blood, two nearly identical Ambion kits for PBMC). The only variable that can be isolated is the use of the mirVana PARIS vs miRNA kit." *
  This study was not a comparison of RNA extraction methods. The aim of this study was to find out whether miR-146a-5p and miR-155-5p expression detected in PBMCs mirrored that quantified in total RNA extracted from whole blood. For this reason two main extraction methods had to be used one for extraction from whole blood and the other from PBMCs. Our results clarified the misconceptions reported in the literature suggesting that the levels of miRNAs detected in whole blood and PBMCs correlate with each other. In fact miR-146a-5p and miR-155-5p are both miRNAs that are detectable in acellular sources such as serum and plasma which again reiterates that the miRNA expression quantified in whole blood would be varied from that of PBMCs.
4. *"Heparin, as a PCR inhibitor, may not be the best anticoagulant for use in qPCR-based studies. No special steps are described for removal of heparin."*
  The use of Heparin in this study was driven by the logic that Heparin is one of the most commonly used anti-coagulants for collecting PBMCs from clinical samples. We reported over 90% efficiency of the PCR reactions demonstrating the fact that the data presented in this study had not been compromised by the use of Heparin. The quality of RNA was also high as the 260/280 ratios were above 1.8 as stated in the manuscript.

Minor points

1. *"There are no error bars to indicate biological or technical variability in Figure 3 or 4. "*
  Error bars have been added to illustrate technical variability in Figure 3 and Figure 4, respectively. As the variability is very small the error bars are hardly visible, hence inserted the sentence “The error bars represent technical variability” in the figure legends.
2. *"The workflow of Figure 1 does not indicate that the right-hand-side pathway involved only PBMC, not whole blood." *
  The workflow of Figure 1 has been modified with an added step to indicate that the right-hand-side pathway involved only PBMCs and not whole blood.
3. *"The PAXgene Blood RNA Tube and the PAXgene Blood miRNA Kit are presented as the "gold standard" in the field, yet the citation is a publication by the company, and it does not mention the miRNA kit. This should be replaced by a more objective assessment."*
  The company reference has been substituted with an original research article by Viprey *et al* (2012) which highlights the reliability and suitability of the whole blood collection method using the PAXgene Blood RNA tubes. It is worth noting that the focus of this report is to evaluate the effect of using total RNAs collected via the PAXgene Blood RNA protocol on miRNA quantification rather than on mRNA expressions.
4. *"More clarity is needed in the use of terms. For example, there may or may not be RNA "expression" differences between genders or cell types. In contrast, differences in RNA quantitation observed upon freezing, or addition of PCR inhibitors, or after purification of the same sample with different kits, are not due to "expression" differences. The underlying expression in the cell remains the same; the ability to characterize it changes."*
  Great attention has been given to using terms with clarity and accuracy in the revised manuscript.  
5. *"Citations of the literature are somewhat thin."*
  In contrast to mRNA research where large pools of literatures have investigated numerous factors that could impact on the outcome of mRNA quantification, the miRNA research is still at its infancy and very few studies have investigated the impact of various total RNA extraction methods on the outcome of miRNA quantification although many articles have been published reporting associations of differential miRNA expression with diagnostic or prognostic status of diseases. This manuscript is particularly aimed to inform the miRNA community that different sources and methods used for extracting total RNAs could have significant impact on the outcome of miRNA quantification.
